# The prevalence and potential associations of anti-drug antibodies against adalimumab in patients with non-infectious uveitis: a cross-sectional study

**DOI:** 10.1186/s12348-025-00567-6

**Published:** 2025-12-25

**Authors:** Ashwin Madhavan, Sophie L. Rogers, Julian J. Bosco, Laura Ross, Priya D. Samalia, Anthony J. Hall, Lyndell L. Lim

**Affiliations:** 1 Department of Surgery, Monash University Faculty of Medicine, Nursing and Health Sciences, Wellington Road Clayton, Victoria, 3800 Australia; 2 Centre for Eye Research Australia, Level 10, 200 Victoria Parade East Melbourne, Victoria, 3002 Australia; 3 Faculty of Medicine, Nursing and Health Sciences, Monash University, Wellington Road Clayton, Victoria, 3800 Australia; 4 The Royal Victorian Eye and Ear Hospital, 32 Gisborne Street East Melbourne, Victoria, 3002 Australia; 5 University of Melbourne, Parkville, Victoria, 3052 Australia; 6 Department of Ophthalmology, Te Whatu Ora Health New Zealand Southern, 201 Great King Street, Dunedin, Otago 9016 New Zealand; 7 Department of Ophthalmology, Alfred Health, 55 Commercial Road Melbourne, Victoria, 3004 Australia

**Keywords:** Anti-drug antibodies, Adalimumab, Non-infectious uveitis, Immunogenicity

## Abstract

**Background:**

Adalimumab is effective in treating non-infectious uveitis (NIU), but some patients develop anti-drug antibodies against adalimumab (ADA-A), which can reduce its effectiveness, resulting in active inflammation and vision loss. We sought to determine the prevalence of ADA-A in adults receiving adalimumab for NIU, and investigate factors associated with the presence of ADA-A.

**Body:**

In this cross-sectional study, eighty-one (46 female) consecutive adult patients receiving adalimumab for NIU at The Royal Victorian Eye and Ear Hospital, and Eye Surgery Associates in Melbourne, Australia were recruited from March 2023 to February 2024 inclusive. The median age of patients was 45 years (range 18, 87) with median disease duration of 5.3 years (range 0.4, 25.3), and median duration of adalimumab therapy of 2.3 years (range 0.2, 13.1). Panuveitis (*N* = 27, 33%) was the commonest anatomical form of uveitis treated, and most patients had bilateral uveitis (*N* = 73, 90%). The most common diagnoses were presumed idiopathic uveitis (*N* = 27, 33%) and sarcoidosis (*N* = 13, 16%). Most patients (*N* = 50, 62%) were concurrently treated with conventional immunosuppression, most commonly using methotrexate (*N* = 32, 40%). ADA-A were present in 5/81 patients (6.2%, 95%CI 2.7, 13.6), and their presence was associated with higher Body Mass Index [median 34.9 kg/m^2^ (IQR 32.5, 38.0) vs. 28.4 kg/m^2^ (IQR 24.4, 31.9), *p* = 0.010], higher C-reactive protein [median 7.4 mg/L (IQR 5.5, 7.9) vs. 2.0 mg/L (IQR 0.0, 6.0), *p* = 0.030], lower patient-reported health [median 5/10 (IQR 5, 6) vs. 8/10 (IQR 6, 8), *p* = 0.024], and lower serum adalimumab levels [median 0.0 µg/mL (IQR 0.0, 0.0) vs. 5.0 µg/mL (IQR 2.8, 7.8), *p* = 0.002]. There was no association between ADA-A and the duration of adalimumab therapy, use of concurrent conventional immunosuppression, presence of systemic inflammatory disease, uveitis activity, visual acuity or adverse effects to adalimumab.

**Conclusion:**

ADA-A were uncommon, and their presence may be associated with obesity, increased C-reactive protein, and poorer patient-reported health. Within the limitations of our statistical power, the presence of ADA-A was not associated with systemic inflammatory disease, uveitis activity, nor adalimumab monotherapy.

## Background

Uveitis is responsible for 10–15% of blindness in the developed world [1]. Non-infectious uveitis (NIU) accounts for 91% of uveitis cases in the United States, and is presumed to be autoimmune in nature [2]. Thus, NIU is treated with corticosteroids, as well as conventional and biologic immunosuppression (such as adalimumab).

Adalimumab (Humira; AbbVie Inc, Illinois, USA) is a fully human monoclonal antibody that targets tumor necrosis factor (TNF). Its use in NIU is supported by clinical trial evidence, most notably the VISUAL I and VISUAL II trials which contributed to its approval by the United States Food and Drug Administration for use in non-infectious intermediate, posterior and panuveitis in 2016 [3, 4]. Despite being effective in treating NIU, some patients develop anti-drug antibodies against adalimumab (ADA-A), which can lead to immunogenic treatment failure: a phenomenon that is also recognized in other adalimumab-treated inflammatory diseases such as rheumatoid arthritis and inflammatory bowel disease [5]. Anti-drug antibodies against adalimumab (ADA-A) can reduce circulating serum levels of adalimumab, and thus decrease the drug’s efficacy [6], and can also increase the risk of adverse effects to adalimumab [7–9].

Despite adalimumab’s relatively standardized dosing (most commonly 40 mg administered subcutaneously every two weeks), the frequency of ADA-A development is widely variable depending on the specific disease it is used to treat, ranging from as low as 15% in ankylosing spondylitis, to as high as 38% in rheumatoid arthritis [10, 11]. A large systematic review by Strand and colleagues investigated the frequency of ADA-A across a spectrum of diseases, and described a wide range of prevalences of ADA-A, ranging from 0 to 54% [5]. The reasons for this variation are poorly understood and may be related to the unique immunological processes involved in each autoimmune disease [11–14]. Therefore, research pertaining to ADA-A in other autoimmune diseases may not be generalizable to NIU. Thus, we performed a cross-sectional study of consecutive adult patients receiving adalimumab for NIU to investigate the real-world prevalence of ADA-A in NIU, and any patient, disease or treatment factors that may be associated with their presence.

## Methods

In this cross-sectional study, consecutive adult patients (age ≥ 18 years) with NIU who were receiving adalimumab (irrespective of duration of treatment), were recruited from the Ocular Immunology Clinic at The Royal Victorian Eye and Ear Hospital, and Eye Surgery Associates Melbourne, Australia, from March 2023 to February 2024 inclusive. The Ocular Immunology Clinic at The Royal Victorian Eye and Ear Hospital is the quaternary referral center for the state of Victoria for patients with uveitis. Eye Surgery Associates is a private practice with four ophthalmologists sub-specializing in uveitis. Written informed consent was obtained from all patients. Patients receiving adalimumab via any dosing regimen, in combination with any local or systemic uveitis treatments were eligible for inclusion in the study. Patients with systemic inflammatory disease in addition to NIU were included. However, patients with a primary diagnosis of an ocular inflammatory disease other than NIU were specifically excluded (e.g. scleritis or infectious uveitis). This study was approved by the Royal Victorian Eye and Ear Hospital Human Research Ethics Committee (reference number 22/1557HL) and was conducted in adherence with the tenets of the *Declaration of Helsinki*.

Each patient underwent venipuncture strictly during the 48-hour trough window prior to a scheduled adalimumab dose, to minimize interference with circulating free drug [15]. All samples were tested for serum adalimumab levels and ADA-A using *i-Tracker Anti-Adalimumab*, a validated chemiluminescent assay at Sullivan Nicolaides Pathology in Bowen Hills, Queensland, Australia [16]. Samples were not batched. Serum adalimumab levels were reported in a range from 0.5 to 24 µg/mL, with levels less than 0.5 µg/mL considered undetectable. ADA-A concentrations greater than 10 ng/mL were considered positive for the presence of ADA-A.

Examination findings were standardized in accordance with the *Standardization of Uveitis Nomenclature (SUN)* criteria [17]. Uveitis was deemed to be active if one or more of the following were present: anterior chamber cells ≥ 1+, vitreous haze ≥ 1+, active retinal or choroidal lesions, active retinal vasculitis, requirement for increased local or systemic treatment for uveitis, or the presence of cystoid macular oedema, defined as a central macular thickness greater than 300 μm in the presence of one or more intra-retinal cystic spaces on spectral-domain optical coherence tomography (SD-OCT, Spectralis; Heidelberg Engineering, Heidelberg, Germany) [18]. Additionally, patients completed a patient-reported outcome questionnaire consisting of questions A1 and A2 from the National Eye Institute Visual Function Questionnaire-25 (VFQ-25), which has been validated in patients with NIU [19, 20]. Questions A1 and A2 were used unchanged from the VFQ-25 to preserve their validity [21].

Clinical data was obtained at the visit that was chronologically closest to the blood draw for ADA-A. Clinical exam was on the same day or within seven days of blood draw in 39/81 (48%) patients, and within twenty-eight days in 69/81 (85%) patients. In patients with bilateral uveitis who had different anatomical locations of uveitis in each eye, the most severe anatomical location in either eye was recorded (with panuveitis regarded as the most severe, followed by posterior, intermediate and anterior uveitis).

### Statistical analysis

Data was de-identified and entered in a purpose-built Research Electronic Data Capture (REDCap) database (REDCap version 13.8.3, Vanderbilt University, Tennessee, USA) and exported into Stata/IC 15.1 for Windows (StataCorp LLC, College Station, Texas, USA).

The primary outcome was the prevalence of ADA-A, calculated as a proportion with a 95% confidence interval calculated using the Wilson score interval method. Secondary outcomes were the potential associations between the presence of ADA-A and patient, disease and treatment characteristics. Categorical variables were compared using Fisher’s exact test. Normally distributed variables were compared using an unpaired t-test, whilst non-parametric variables were compared using the Wilcoxon rank-sum test. Results were considered statistically significant when *p*-values were less than 0.05.

## Results

### Patient and treatment characteristics

Of the 81 patients recruited, 46/81 (57%) patients were female, and the median age was 45 years (range 18, 87) (Table 1). The most common anatomical location was panuveitis (*N* = 27, 33%), while the most common etiologies were presumed idiopathic (*N* = 27, 33%), sarcoidosis (*N* = 13, 16%) and Behçet’s disease (*N* = 8, 10%) (Table 1).

**Table 1:** Baseline characteristics of patients treated with adalimumab (*N* = 81)

Characteristic	Study cohort (*N* = 81)	
Age, median (IQR), years	45.0 (33.0, 52.0)	
Sex, number of females (%)	46 (57)	
Ethnicity, n (%)	European	59 (73)
	Asian	14 (17)
	Middle Eastern	4 (5)
	African	2 (2)
	American	1 (1)
	Unknown	1 (1)
Body Mass Index (BMI)^a^, median (IQR), kg/m^2^	29.2 (24.7, 33.3)	
Body Mass Index (BMI)^a^, n (%)	BMI < 25 kg/m^2^	13 (27)
	Overweight (BMI 25–29.9 kg/m^2^)	12 (25)
	Obese (BMI 30 kg/m^2^ or higher)	23 (48)
Anatomic location of uveitis, n (%)	Anterior	19 (24)
	Intermediate	18 (22)
	Posterior	17 (21)
	Panuveitis	27 (33)
Bilateral uveitis, n (%)	73 (90)	
Systemic inflammatory disease, n (%)	39 (48)	
Duration of uveitis, median (IQR), years	5.3 (2.7, 7.6)	
Etiological diagnosis, n (%)	Idiopathic	27 (33)
	Sarcoidosis	13 (16)
	Behçet’s disease	8 (10)
	Vogt-Koyanagi-Harada disease	6 (7)
	Birdshot Chorioretinopathy	6 (7)
	HLA-B27 associated	5 (6)
	Tattoo associated uveitis	4 (5)
	Multifocal choroiditis	3 (4)
	Juvenile Idiopathic Arthritis associated	2 (3)
	Punctate inner choroidopathy	2 (3)
	Tubulointerstitial nephritis and uveitis	2 (3)
	Serpiginous choroiditis	1 (1)
	Sympathetic ophthalmia	1 (1)
	Inflammatory Bowel Disease associated	1 (1)

^a^BMI data was available in 48/81 (59%) of patients

The most common dosing regimen was adalimumab 40 mg (Humira; AbbVie Inc, Illinois, USA) administered subcutaneously every two weeks (*N* = 65, 80%). Median treatment duration was 2.3 years (range 0.2, 13.1), and most patients (*N* = 50, 62%) received concurrent treatment with conventional synthetic disease-modifying anti-rheumatic drugs (cDMARDs), most commonly methotrexate (*N* = 32, 40%), mycophenolate (*N* = 10, 12%) and azathioprine (*N* = 5, 6%). Many patients were also receiving concurrent oral corticosteroids (*N* = 21, 26%) and topical corticosteroids (*N* = 25, 31%) (Table 2). Side effects to adalimumab were uncommon (*N* = 4, 5%), consisting of injection site reaction (*N* = 1), persistent cough (*N* = 1), maculo-papular rash (*N* = 1), and unspecified (*N* = 1). One patient had previously ceased adalimumab therapy in the setting of cutaneous melanoma and subsequently resumed adalimumab therapy following formal oncological treatment.

**Table 2:** Treatment characteristics of patients receiving adalimumab (*N* = 81)

Characteristic	Study cohort (*N* = 81)	
Duration on adalimumab, median (IQR), years	2.3 (1.1, 3.7)	
Adalimumab dosing regimen, n (%)	80 mg every week	1 (1)
	40 mg every week	4 (5)
	40 mg every 2 weeks	65 (80)
	40 mg every 3 weeks	8 (10)
	40 mg every 4 weeks	3 (4)
Break in adalimumab therapy in last 3 months, n (%)	14 (17)	
Side effects to adalimumab, n (%)	4 (5)	
Any concurrent cDMARD use, n (%)	50 (62)	
Concurrent cDMARD used^a^, n (%)	Methotrexate	32 (40)
	Mycophenolate	10 (12)
	Azathioprine	5 (6)
	Tacrolimus	2 (2)
	Hydroxychloroquine	2 (2)
Concurrent oral steroid use, n (%)	21 (26)	
Concurrent topical steroid use, n (%)	25 (31)	
Total number of steroid-sparing agents used at any time, median (IQR)	3 (IQR 2, 3)	
Ocular steroid injections in last 3 months, n (%)	0 injections	74 (91)
	1 injection	7 (9)
Concurrent topical intra-ocular pressure-lowering drop use, n (%)	20 (25)	
Past use of biologic immunosuppression for uveitis, n (%)	2 (3)	

^a^Some patients received more than one concurrent cDMARD

### Patients with anti-drug antibodies against adalimumab

ADA-A were present in 5/81 patients (6.2%, 95%CI 2.7, 13.6) (Table 3). In four out of these five patients with ADA-A, serum adalimumab levels were undetectable. Quantitative ADA-A levels ranged from 15 ng/mL to 963 ng/mL, with a median ADA-A level of 188 ng/mL. Etiologically, three of these five patients had presumed idiopathic uveitis, while the remaining two patients had Vogt-Koyanagi-Harada Disease. Three out of these five patients were receiving a concurrent cDMARD (two receiving methotrexate and one receiving tacrolimus), and two out of five patients were deemed to have active uveitis at the time of ADA-A testing (Table 3).

**Table 3:** Clinical characteristics of each patient with ADA-A (*N* = 5)

Age / Sex	Etiology	Laterality and anatomical location	Years of disease	Years of adalimumab therapy	Adalimumab dosing		VA logMAR (Snellen)	Activity	Topical steroid frequency	Concurrent cDMARD	PNL Dose	Clinical outcome	Serum drug level (µg/mL)	ADA-A level (ng/mL)
24M	Idiopathic	Right Panuveitis	6.6	4.8	40 mg every two weeks	**OD**	0.48 (20/60)	**Active**	TDS	MTX	-	Switched to interleukin-6 inhibitor	< 0.50	188
						**OS**	0 (20/20)	Quiet	-					
49F	VKH	Bilateral Posterior	1.9	1.9	40 mg every two weeks	**OD**	0.10 (20/25)	Quiet	-	-	-	Ceased adalimumab	< 0.50	75
						**OS**	0 (20/20)	Quiet	-					
55F	Idiopathic	Right Anterior	7.5	0.9	40 mg every two weeks	**OD**	0.30 (20/40)	Quiet	BD	MTX	-	Continued adalimumab	3.8	15
						**OS**	-0.08 (20/17)	Quiet	-					
56F	Idiopathic	Bilateral Intermediate	8.1	6.2	40 mg every two weeks	**OD**	0.80 (20/125)	Quiet	Q2H	TAC	5 mg	Ceased adalimumab	< 0.50	963
						**OS**	1.40 (20/500)	Quiet	QID					
65M	VKH	Bilateral Panuveitis	3.3	1.8	40 mg every two weeks	**OD**	0.30 (20/40)	**Active**	-	-	2 mg	Continued adalimumab	< 0.50	393
						**OS**	0 (20/20)	Quiet	-					

Abbreviations: **ADA-A** = anti-drug antibodies against adalimumab; **BD** = twice daily; **cDMARD** = conventional synthetic disease-modifying anti-rheumatic drug; **MTX** = methotrexate; **OD** = right eye; **OS** = left eye; **Q2H** = every 2 h; **QID** = four times daily; **PNL** = prednisolone; **TAC =** tacrolimus; **TDS** = three times daily; **VA** = Visual Acuity; **VKH** = Vogt-Koyanagi-Harada disease

**Fig. 1:**
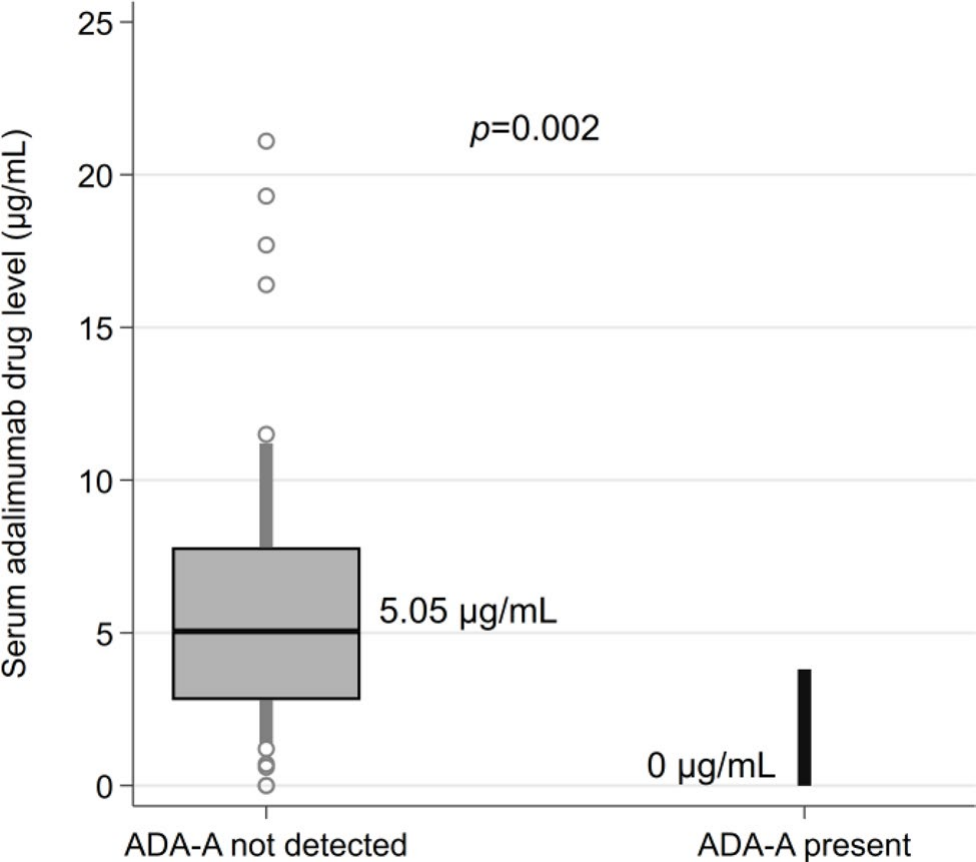
Adalimumab levels in patients without ADA-A (*N* = 76) and with ADA-A (*N* = 5). Boxes represent the median and interquartile range, whilst whiskers indicate the 10th and 90th percentiles. Values outside of these percentiles are plotted as circles. Median drug levels for each group are reported along with the *p*-value from a Wilcoxon Rank Sum test to compare the groups

In comparison to patients without ADA-A, patients with ADA-A had lower serum adalimumab levels [median 0.0 µg/mL (IQR 0.0, 0.0) vs. 5.0 µg/mL (IQR 2.8, 7.8), *p* = 0.002] (Fig. 1). Additionally, patients with ADA-A had higher Body Mass Index [median 34.9 kg/m^2^ (IQR 32.5, 38.0) vs. 28.4 kg/m^2^ (IQR 24.4, 31.9), *p* = 0.010] and were more likely to be obese [5/5 (100%) vs. 18/76 (42%), *p =* 0.020] (Table 4).

As a non-specific marker of systemic inflammation, C-reactive protein [22] was higher in patients with ADA-A compared to patients without ADA-A [median 7.4 mg/L (IQR 5.5, 7.9) vs. 2.0 mg/L (IQR 0.0, 6.0), *p* = 0.030]. We also found that when asked Question A1 from the Visual Function Questionnaire-25, patients with ADA-A reported lower overall health compared to patients without ADA-A [median 5/10 (IQR 5,6) vs. median 8/10 (IQR 6,8), *p* = 0.024] (Table 4). Questionnaires were completed at the time of recruitment, prior to knowledge of ADA-A presence. We observed no statistically significant differences in age, sex, duration of adalimumab therapy, use of a concurrent cDMARD or the presence of systemic inflammatory disease. There were no statistically significant differences in uveitis activity at the time of testing, visual acuity or side effects to adalimumab (Table 4).

**Table 4:** Outcomes in patients without ADA-A (*N* = 76) and with ADA-A (*N* = 5)

Parameter	*No ADA-A* *N* = 76	*ADA-A* *N* = 5	*p*-value
Age in years, median (IQR)	44.0 (32.0, 52.0)	55.0 (49.0, 56.0)	0.198
Sex, n female (%)	43 (57)	3 (60)	1.000
Body Mass Index (BMI)^a^ in kg/m^2^, median (IQR)	28.4 (24.4, 31.9)	34.9 (32.5, 38.0)	**0.010**
Body Mass Index^a^, N (%) BMI < 25 kg/m^2^ Overweight (BMI 25 to 29.9 kg/m^2^) Obese (BMI 30 kg/m^2^ or higher)	13 (30) 12 (28) 18 (42)	0 0 5 (100)	0.067
Obese^a^ (BMI ≥ 30 kg/m^2^), N (%)	18 (42)	5 (100)	**0.020**
Bilateral uveitis, N (%)	70 (92)	3 (60)	0.074
Years of uveitis, median (IQR)	5.2 (2.6, 7.7)	6.4 (3.0, 7.4)	0.784
C-reactive protein^b^ in mg/L, median (IQR)	2.0 (0.0, 6.0)	7.4 (5.5, 7.9)	**0.030**
Use of concurrent cDMARD, N (%)	47 (62)	3 (60)	0.935
Use of concurrent oral steroids, N (%)	19 (25)	2 (40)	0.600
Years of adalimumab therapy, median (IQR)	2.3 (1.0, 3.7)	1.9 (1.6, 4.7)	0.858
Presence of systemic inflammatory disease, N (%)	36 (48)	2 (40)	1.000
Serum adalimumab level in µg/mL, median (IQR)	5.0 (2.8, 7.8)	0.0 (0.0, 0.0)	**0.002**
Breaks in adalimumab in past 3 months, N (%)	12 (16)	2 (40)	0.209
Active uveitis, N (%)	21 (28)	2 (40)	0.619
Visual acuity (logMAR), median (IQR)	0.0 (-0.1, 0.1)	0.0 (0.0, 0.0)	0.545
Side effects to adalimumab, N (%)	5 (6.6)	0 (0)	1.000
A1: How would you rate your overall health, on a scale where zero is as bad as death, and 10 is best possible health? Median (IQR)	8/10 (6, 8)	5/10 (5, 6)	**0.024**
A2: How would you rate your eyesight now (with glasses or contact lens on, if you wear them), on a scale of from 0 to 10, where zero means the worst possible eyesight, as bad or worse than being blind, and 10 means the best possible eyesight? Median (IQR)	7/10 (6, 8)	7/10 (5, 7)	0.497

^a^BMI data was present in 48/81 (59%) patients, including 5/5 patients with ADA-A, and 43/76 without ADA-A

^b^C-reactive protein data was present in 57/81 (70%) patients, including 4/5 patients with ADA-A, and 53/76 without ADA-A

## Discussion

Our key finding was a low prevalence of ADA-A, present in 6.2% of patients. In their systematic review, Fernández-Nogueras found a wide range of frequency of ADA-A in NIU from 2.7% to 45.0% [23]. As acknowledged by the authors, a limitation of retrospective studies remains the selection bias towards preferentially testing patients who are suspected of failing adalimumab therapy, thus manifesting a higher pre-test probability of finding ADA-A. Although the low rates of ADA-A in the VISUAL I (2.7%) and VISUAL II (5.2%) trials may be attributable to the fact that not all patients were tested for ADA-A [23], our prevalence of 6.2% from testing 81 consecutive patients irrespective of their clinical uveitis activity suggests that the real-world prevalence of ADA-A in NIU may indeed be low.

This low frequency of ADA-A supports the Pachón-Suárez review [24], and raises the possibility that ADA-A may occur less frequently in NIU compared with inflammatory diseases in other parts of the body [5]. Interestingly, Cordero-Coma et al. found that patients with systemic inflammatory disease in addition to uveitis were at higher risk of developing ADA-A, compared to patients with isolated uveitis [25], though we did not observe this association. This may be due to differences in the representation of systemic inflammatory diseases, with sarcoidosis being the most common in our study, compared with spondyloarthropathies in the Cordero-Coma study, and it is possible that certain inflammatory diseases carry a greater predisposition for ADA-A development [25].

Interestingly, we found that patients with ADA-A had higher C-reactive protein levels compared to patients without ADA-A (Table 3). With C-reactive protein being a non-specific serum marker of systemic inflammation [22], it is unclear whether increased systemic inflammation predisposes to ADA-A development or conversely, whether it is the presence of ADA-A that results in decreased capture of tumor necrosis factor, ultimately resulting in increased systemic inflammation. In any case, we advise caution when interpreting this association with C-reactive protein, given that patients with ADA-A also had higher BMI compared to patients without ADA-A. All patients with ADA-A were obese (BMI ≥ 30 kg/m^2^), and this chronic inflammatory state can independently cause C-reactive protein to be elevated [26], though we are unable to perform multivariate analysis to adjust for this due to the overall low frequency of ADA-A.

The role of obesity in adalimumab treatment failure has been well described in other autoimmune diseases such as rheumatoid arthritis, psoriatic arthritis and inflammatory bowel disease [27, 28]. A large meta-analysis of 54 rheumatological studies revealed that obesity increases the risk of treatment failure in these conditions by up to 80%, with each 1 kg/m^2^ increase in BMI conferring a 6.5% higher risk of failing anti-TNF therapy [27]. Similarly in NIU, Pichi and colleagues described obesity as a predictor of non-response to adalimumab [29]. Our results support this association, with the caveats noted above. Given that all of our patients who developed ADA-A were obese, and the fact that patients with ADA-A had higher BMI than patients without ADA-A (Table 4), it is possible that obesity increases the risk of immunogenicity against adalimumab in patients with NIU. It should also be noted that almost half of our cohort (48%) were obese (Table 1). As weight gain is an established side effect of systemic corticosteroids [30], and over a quarter of our patients were indeed being treated with concurrent systemic corticosteroids (Table 2), it is highly likely that we are contributing to these high rates of obesity in this patient population.

One universal limitation encountered while studying ADA-A is the lack of a globally standardized assay. There are numerous assay techniques available, which vary in their ability to discern clinically significant neutralizing ADA-A [31]. This can result in variations in the rate of ADA-A detected across studies. As an example, in their study of 80 patients with NIU, Pichi et al. found 51.2% of patients to have ADA-A [29], compared with 6.2% in our study of 81 patients. Although it is unusual to encounter such a high rate of ADA-A, it raises a critical point regarding the inherent variation in commercially available assays with respect to their methodology, standardization, and tolerance of circulating free drug: all of which can influence an assay’s sensitivity in detecting ADA-A [16, 31]. We used a standardized, validated chemiluminescent assay at a single laboratory (allowing for routine changes in assay and reagent batches), in attempt to overcome the heterogeneity in techniques used across previous retrospective studies in NIU [24, 32–34]. The high ADA-A levels and corresponding undetectable drug levels suggest that ADA-A present were neutralizing ADA-A, reinforcing the validity of our assay in detecting clinically significant ADA-A (Fig. 1) [35]. Moreover, our high rate of concurrent cDMARD use (*N* = 50, 62%) may have partly contributed to our low rate of ADA-A. While we were not able to determine whether concomitant cDMARD use was protective within the limitations of our statistical power, the use of a concomitant cDMARD is a well-studied modifiable factor that has been associated with decreased risk of ADA-A in numerous studies, most notably in the prospective studies of 272 patients with rheumatoid arthritis by Bartelds [6], and 174 patients with Crohn’s disease by Vermeire [36]. In NIU, the retrospective studies by Bellur, Leinonen and Eurelings suggest that the concurrent use of a cDMARD may similarly be protective against ADA-A, with the anti-metabolites methotrexate and mycophenolate being the most commonly studied agents [15, 37, 38]. Calcineurin inhibitors such as tacrolimus and ciclosporin may also prevent ADA-A formation, though they are typically regarded as providing mainly T cell immunosuppression, compared with anti-metabolites such as methotrexate which have a dual action on both T and B cells [39, 40]. Thus, calcineurin inhibitors may theoretically have less impact on the antibody response compared with anti-metabolites, though this has not been clearly established in the literature around NIU nor other rheumatological conditions or inflammatory bowel disease. In our cohort, the low number of patients with ADA-A coupled with the low numbers with concomitant calcineurin inhibitor use limited our ability to compare this phenomenon between classes of cDMARDs.

Adalimumab dosing regimens may also be implicated in the development of ADA-A. In a small retrospective case-control study of 12 NIU patients with ADA-A, Bromeo and colleagues found that weekly dosing of adalimumab conferred a lower risk of ADA-A development compared to standard fortnightly dosing [41]. We did not observe such a correlation in our study, perhaps due to the small number of patients who were receiving weekly dosing (*N* = 5, 6%), compared with the majority receiving standard fortnightly dosing (*N* = 65, 80%). Another possible contributing factor is patient compliance with adalimumab injections, given that missing doses for any reason has been associated with increased risk of ADA-A development [42, 43]. In our study, only 14/81 (17%) patients had missed one or more doses of adalimumab in the three months prior to testing, usually due to a doctor-advised reason (e.g. infection) or a delay in receiving medication from their pharmacy (Table 2). It is possible that poor compliance to adalimumab therapy in other studies may explain their higher rates of ADA-A, although this is difficult to assess retrospectively. Similarly, the use of biosimilar medications may lead to variations in ADA-A formation, though there is evidence to suggest that adalimumab biosimilar medications have a similar immunogenicity profile to the reference product adalimumab [44, 45]. All 81 patients in our study received adalimumab in the form of Humira (AbbVie Inc, Illinois, USA).

With respect to the impact of ADA-A on clinical disease activity, we observed a trend towards a higher proportion of patients with ADA-A having active uveitis, but this did not reach statistical significance [2/5 (40%) vs. 21/76 (28%), *p* = 0.619]. Future larger studies that are suitably powered to detect differences in uveitis activity according to the presence of ADA-A are required to further investigate this possible association. Despite no difference in uveitis activity or visual acuity, it was interesting to see that patients with ADA-A reported worse overall health compared to patients without ADA-A [5/10 (IQR 5, 6) vs. 8/10 (IQR 6, 8), *p* = 0.024] (Table 3). This may be attributed to other ocular symptoms that affect quality of life but are not captured by visual acuity alone, such as eye pain, glare and photophobia [19, 46]. Moreover, the association we have found between obesity and ADA-A may also be contributory, given the numerous health consequences of obesity and metabolic syndrome [47, 48]. In rheumatoid arthritis, low adalimumab drug levels have been associated with worse Health Assessment Questionnaire-Disability Index (HAQ-DI) scores [49], but to our knowledge, this association between ADA-A and poorer patient-reported health is a novel finding.

The major strengths of our study include our recruitment of consecutive patients and large sample size, all of which enabled a closer approximation of the real-world prevalence of ADA-A in NIU. By recruiting consecutive patients receiving adalimumab, we sought to minimize selection bias, compared with retrospective studies that were performed selectively in patients suspected of failing adalimumab therapy, which may artificially raise the pre-test probability of finding ADA-A. Moreover, by recruiting patients at a quaternary referral center for uveitis, we believe our diverse patient cohort is representative of adult patients with NIU requiring treatment with adalimumab, comprising 14 different uveitides (Table 1). As aforementioned, another key strength of our study was the uniform testing for ADA-A in all patients, compared with the inherent heterogeneity in testing techniques that was naturally encountered in previous retrospective studies [24, 32–34]. Our use of a consistent chemiluminescent assay methodology that is readily available to clinicians, supports the applicability of our results to real-world uveitis practice [16, 34].

We acknowledge several important limitations. While the low prevalence of ADA-A is reassuring, it limited our statistical power to further investigate the factors and clinical impact associated with the presence of ADA-A. Specifically, we were unable to perform multivariate analysis to study the relationship between varying dosing regimens and the rate of ADA-A formation (e.g. adalimumab dosed every three or four weeks compared with fortnightly dosing), and also were unable to further study any associations with concomitant cDMARD usage (including comparison of classes of cDMARDs, e.g. anti-metabolites vs. calcineurin inhibitors in preventing ADA-A formation). With only five patients having developed ADA-A, we were also unable to perform multivariate analysis to control for specific patient characteristics such as BMI which, as an example, can independently increase CRP levels [26]. Moreover, not all patients had BMI data available. With only 48/81 (59%) of patients in our cohort having BMI data, it is quite possible that our analysis around obesity will have suffered from selection bias, where body weight was more likely to be measured for patients in whom obesity was suspected. Additionally, the cross-sectional study design did not allow us to comment on whether ADA-A detected were permanent or transient [50]. It is also possible that some patients who developed ADA-A may not have been enrolled if they ceased adalimumab therapy prior to study commencement, which may have led to selection bias through omission of these cases, and possibly resulted in an underestimate of the prevalence of ADA-A.

## Conclusions

We performed a cross-sectional study of 81 consecutive patients receiving adalimumab for NIU and found ADA-A to be relatively uncommon. The presence of ADA-A may be associated with obesity, increased C-reactive protein, poorer patient-reported health, and reduced serum adalimumab levels.

## Data Availability

The datasets generated and/or analysed during the current study are not publicly available due to privacy concerns, but are available from the corresponding author on reasonable request.

## Abbreviations

ADA-A: Anti-drug antibodies against adalimumab
BD: Twice daily
cDMARD: Conventional synthetic disease-modifying anti-rheumatic drug
HLA-B27: Human leukocyte antigen B27
IQR: Inter-quartile range
MTX: Methotrexate
NIU: Non-infectious uveitis
OD: Right eye
OS: Left eye
PNL: Prednisolone
Q2H: Every 2 h
QID: Four times daily
REDCap: Research Electronic Data Capture
SUN: Standardization of Uveitis Nomenclature
TAC: Tacrolimus
TDS: Three times daily
VISUAL trials: Efficacy and Safety of Adalimumab in Patients With Active Uveitis
VFQ-25: National Eye Institute Visual Function Questionnaire-25
VKH: Vogt-Koyanagi-Harada disease
